# Revealing the Active Role of the Gate Electrode in Weak-Light Detection

**DOI:** 10.1021/acsnano.5c13029

**Published:** 2026-03-05

**Authors:** Tzu-En Huang, Chen-Yu Wang, Hua-Hsing Liu, Bor-Wei Liang, Shih-Chia Peng, You-Jia Huang, Yann-Wen Lan, Kuan-Ming Hung, Kuang Yao Lo

**Affiliations:** 1 Department of Physics, 34912 National Cheng Kung University, Tainan 701, Taiwan; 2 Institute of Electro-Optical Engineering, 34879 National Taiwan Normal University, Taipei 11677, Taiwan; 3 Department of Physics, 34879 National Taiwan Normal University, Taipei 11677, Taiwan; 4 Department of Electronic Engineering, 517768 National Kaohsiung University of Science and Technology, Kaohsiung 807, Taiwan

**Keywords:** negative photocurrent, weak-light photodetection, gate-induced injection, MoS_2_ FET, interface traps, substrate-mediated effects

## Abstract

The contribution of gate materials to the photoresponse of thin-film field-effect photodetectors has long been overlooked, with prior studies focusing primarily on photoconductive and photogating effects within the sensing layer. Here, we show that under weak illumination, photocarriers primarily originate from the silicon gate rather than the MoS_2_ channel. Absorption spectra confirm that light is mainly absorbed by the gate, driving a negative photocurrent (NPC). The NPC magnitude and slope vary with illumination intensity and *V*_DS_, suggesting transport dominated by Si/SiO_2_ interface traps. NPC persists when MoS_2_ is replaced with Au/Ti, reinforcing the gate-driven mechanism. At higher powers, band bending reverses due to competing photovoltaic and trap-induced potentials. These results highlight the active role of the gate and offer strategies for device optimization.

## Graphical abstract

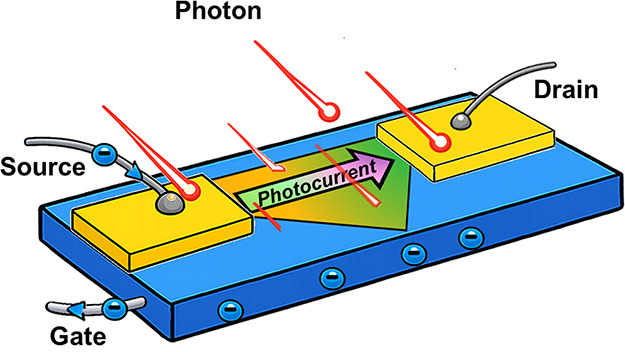

## 1 Introduction

Weak light detection is a critical capability for next-generation optoelectronic systems, with applications ranging from ultralow light imaging to quantum photonics and biosensing.^1–3^ Two-dimensional (2D) semiconductors such as monolayer MoS_2_ are particularly attractive for these applications due to their layer-dependent bandgap,^4–6^ ultrathin geometry,^7,8^ and gate-tunable electronic properties.^9–12^ Previous studies have shown that under strong illumination, photoconductive (PC) and photovoltaic (PV) effects within the MoS_2_ channel dominate the photocurrent response.^9,13–16^ However, under weak optical excitation, where the contribution from conventional PC and PV mechanisms are relatively weak, alternative photoresponse pathways—such as trap-assisted gain and contact- or gate-related effects—can become significant.^17,18^ These mechanisms remain underexplored and are often overlooked in standard interpretations of 2D phototransistor behavior.

In this work, we examine a configuration where the gate is electrically connected to the source terminal (*V*_G_ = 0 V), forming a closed conduction loop. This configuration is distinct from a floating gate, which lacks a defined potential and does not support direct charge transfer. We demonstrate that, under weak illumination, photocarriers generated in the gate material can contribute in the device’s photocurrent, revealing a nontrivial contribution that is typically underestimated.

Notably, this gate-assisted photocurrent under weak illumination presents a new opportunity to enhance detection sensitivity without external gain circuitry. By exploiting charge transfer mechanisms across the dielectric, it becomes possible to extract meaningful signal from illumination intensities in the nanowatt-per-square-centimeter range. In this work, we systematically examine this behavior and provide evidence for a gate-driven photocurrent component that is electrically active only under gate-source connectivity, establishing a new perspective for enhancing sensitivity in 2D optoelectronic sensors.

The MoS_2_-based FET utilized in this study was synthesized via chemical vapor deposition (CVD). A detailed description of the fabrication steps is provided in the Supporting Information S1. Figure 1a presents a schematic illustration of the device structure, while Figure 1b shows an optical microscope image of the fabricated sample. Figure 1c,d compare the photoresponse of a monolayer MoS_2_ FET photodetector under two configurations: with the gate-source electrodes connected at *V*_G_ = 0 V (Figure 1c) and disconnected (Figure 1d). When connected, the device exhibits a clear intensity-dependent photocurrent under 460 nm illumination, while the disconnected configuration shows negligible response. This contrast highlights the active role of the gate material in photodetection, emphasizing its significance in the design and analysis of phototransistors.

**Figure 1:**
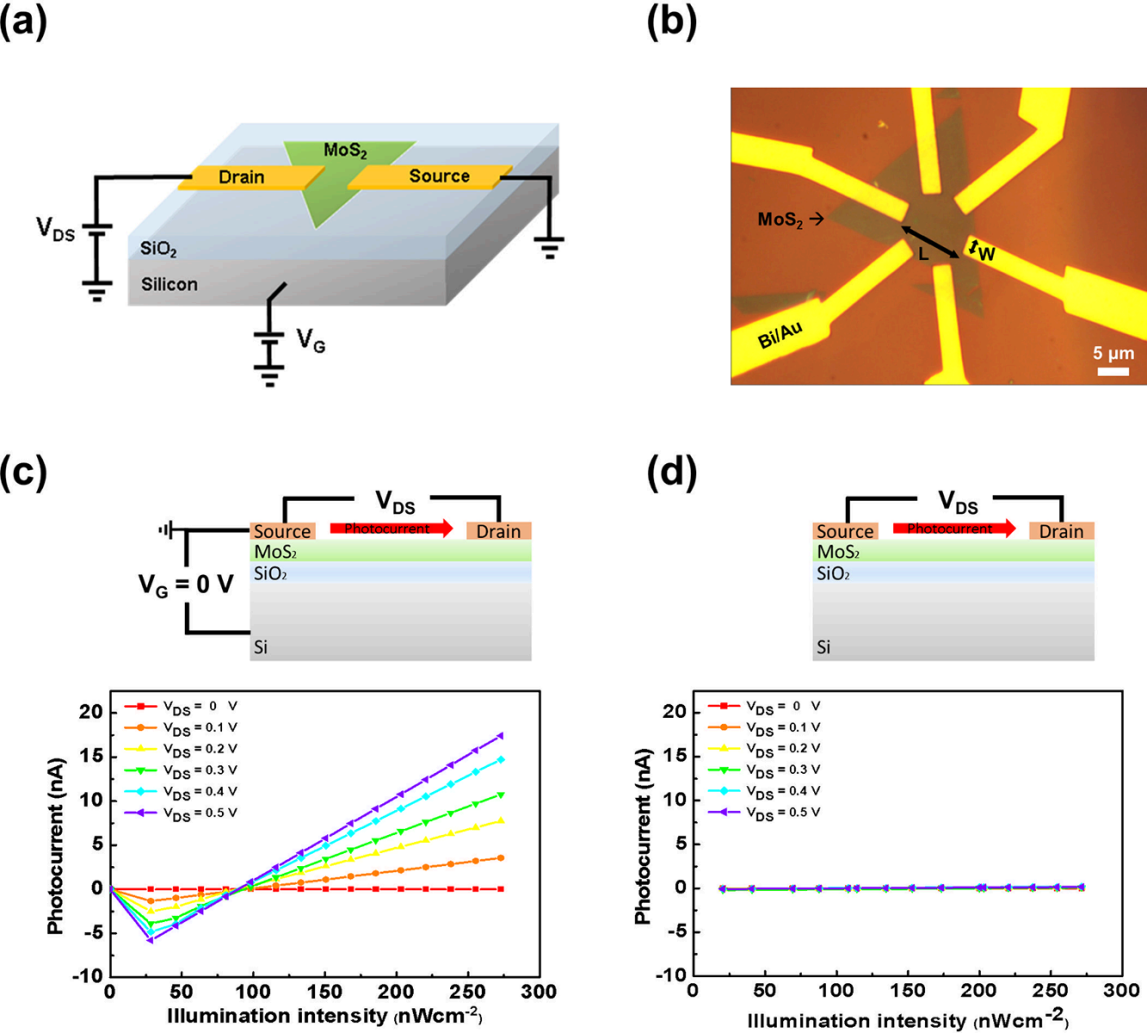
(a) Schematic illustration of the MoS_2_-based photodetector. (b) Optical microscope image of the fabricated monolayer MoS_2_ photodetector device. (c) Photocurrent response of a monolayer MoS_2_-based field-effect transistor under illumination from a 460 nm light source at varying optical intensities, with the gate-source electrodes connected. (d) Photocurrent response of the same device under identical illumination conditions, but without the gate-source electrodes connection.

## 2 Results and Discussion

### 2.1 Theory

The optical absorbance of the FET’s two main structural components—Si and MoS_2_—was calculated across the visible spectrum (Figure 2a), showing good agreement with literature values.^19,20^ Owing to its atomically thin profile, MoS_2_ exhibits limited optical absorption, resulting in most incident photons being absorbed by the underlying silicon gate rather than the MoS_2_ layer.

**Figure 2:**
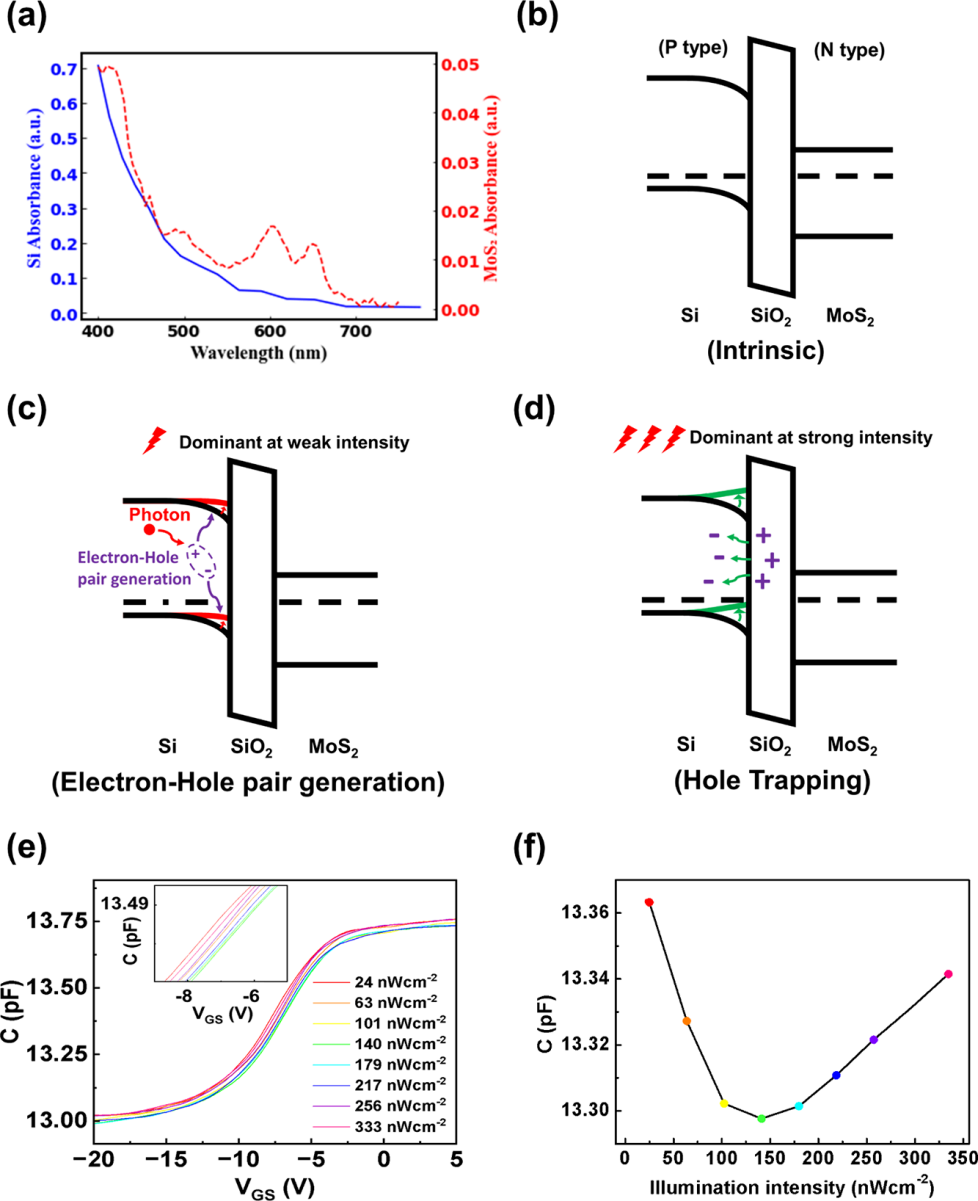
(a) Optical absorbance spectra of Si (blue curve) and MoS_2_ (red curve), highlighting the difference in absorption efficiency between the two materials. (b) Intrinsic band diagram of the Si/SiO_2_/MoS_2_ heterostructure before illumination, illustrating the initial energy band alignment. (c) Band diagram of the Si/SiO_2_/MoS_2_ heterostructure under illumination, in the absence of a gate-source connection, showing the photogenerated carrier distribution. (d) Band diagram of the Si/SiO_2_/MoS_2_ heterostructure under illumination with a gate-source connection, demonstrating the charge transfer mechanism. (e) Smoothed capacitance–voltage curves under 590 nm illumination at varying intensities. (f) Capacitance versus illumination intensity at *V*_G_ = −9 V, showing a decrease-then-increase trend due to competing effects of photovoltage and trap ionization.

Another critical factor to consider is the internal quantum efficiency (IQE) of the two materials. Previous studies have reported that the IQE of monolayer MoS_2_ is approximately 8%,^21^ whereas Si can achieve values as high as 85%.^22^ This significant contrast suggests that, in MoS_2_-based FET devices under illumination, the majority of photogenerated charge carriers are primarily generated in the gate material (Si). Due to the electrical connection between the gate and source electrodes, these photogenerated electrons can subsequently transfer into the MoS_2_ layer, effectively influencing its optoelectronic response. This mechanism highlights the crucial role of the gate material in the overall photocurrent generation of phototransistors.

The charge separation caused by band bending and the capture of holes by interface traps play a dominant role in determining the distribution of photoinduced carriers. Figure 2b illustrates the band diagram of the device when the gate is floating and under dark conditions. Due to the p-type characteristics of Si and the n-type characteristics of MoS_2_, the energy bands of Si bend downward, creating a built-in potential near the Si/SiO_2_ interface. In this circumstance, hole traps below the Fermi level are neutralized by capturing free electrons.

Under illumination, two distinct light absorption processes occur: (i) bulk Si absorbs photons, generating electron–hole pairs, and (ii) interface hole traps absorb photons, generating free electrons while leaving positively ionized traps. In the first case, photogenerated electron–hole pairs in Si are separated by the built-in electric field, producing a photovoltaic voltage that opposes the built-in potential, thereby reducing it, as shown in Figure 2c. In the second case, ionization of hole traps leads to the release of free electrons, generating a trap-induced potential that enhances the built-in potential, as shown in Figure 2d. The interplay between these two competing mechanisms results in a transition of the energy band bending from downward to upward as the illumination intensity increases.

It is important to note that bulk Si, having a three-dimensional lattice structure, exhibits higher light absorption efficiency compared to the two-dimensional hole traps at the SiO_2_ interface. Consequently, at low illumination intensity, the photovoltaic effect dominates, leading to the accumulation of positive charge in the bulk Si. As the light intensity increases, the growing photovoltage initially opposes the built-in potential. At this stage, the potential generated by ionized hole traps starts to dominate, driving the energy band to bend upward and altering the overall polarity of the potential at the Si/SiO_2_ interface. This results in the accumulation of negative charge in the bulk Si. Similar behavior has been observed in previous researches.^17^

Capacitance–voltage (CV) measurements were performed on our sample to probe the interfacial behavior under illumination. Figure 2e displays the smoothed CV curves of the device under varying intensities of 590 nm illumination, additional details of the smoothing procedure are provided in the Supporting Information S3. Under weak illumination, electron–hole pairs are generated within the Si substrate, which reduces the built-in potential and consequently lowers the capacitance, as illustrated in the inset of Figure 2e. As the illumination intensity increases, additional free electrons are released due to the ionization of hole traps, which enhances the built-in potential and results in an increase in capacitance—again evident in the inset of Figure 2e. The horizontal shift of the CV curves can be interpreted as variations in threshold voltage (*V*_th_) as shown in Figure 3a and flat-band voltage (*V*_fb_), with rightward shifts indicating increases in these parameters. Figure 2f further tracks the capacitance at a fixed gate bias (*V*_G_ = −9 V), where a nonmonotonic trend is observed: an initial decrease in capacitance followed by a subsequent increase. This behavior confirms the competing effects of reduced and enhanced built-in potential as illumination intensity rises.

**Figure 3:**
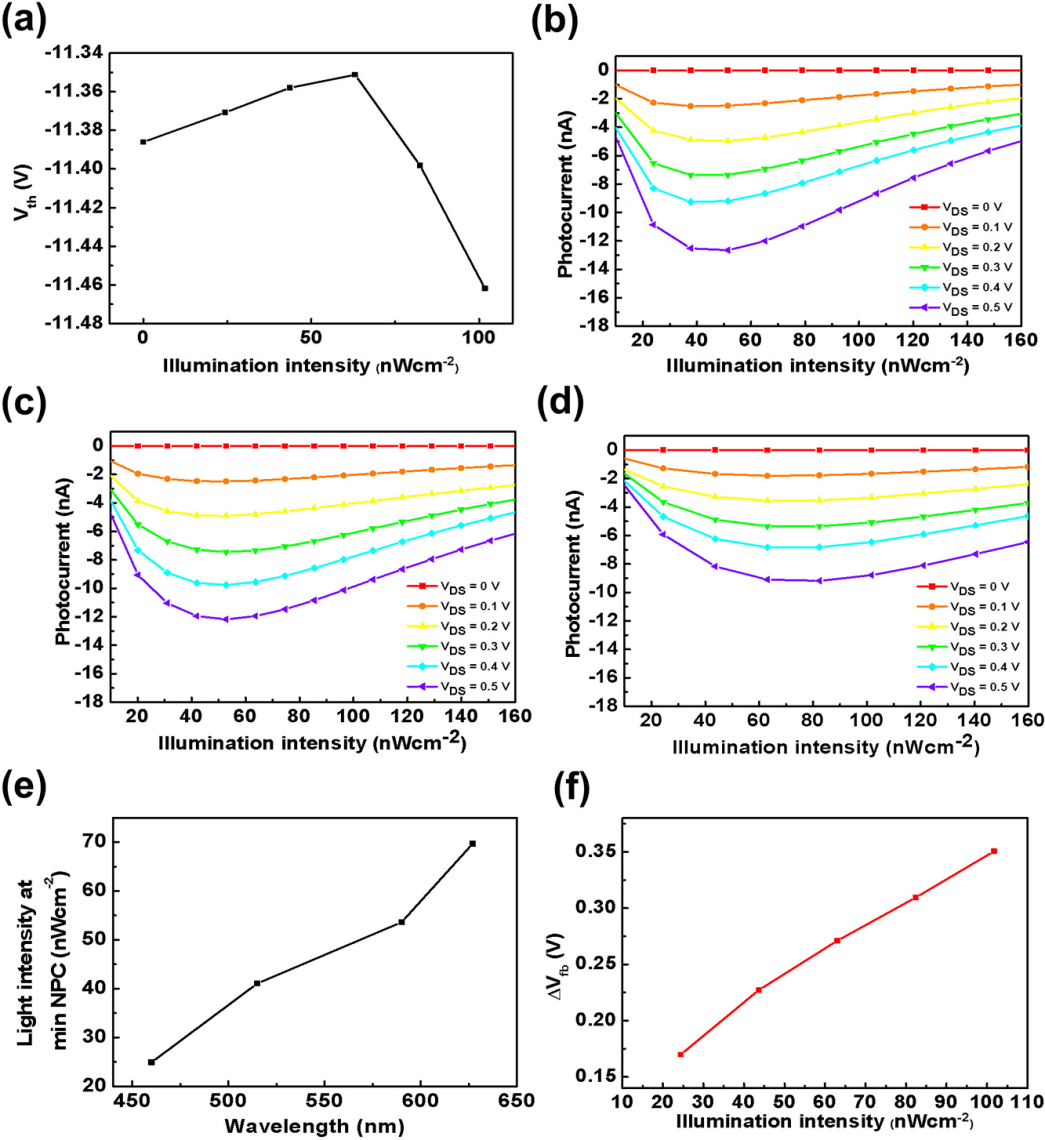
(a) Threshold voltage variation of the MoS_2_ phototransistor under different illumination intensities. (b–d) Photocurrent response of the device under different *V*_DS_ values when illuminated with (b) 515 nm, (c) 590 nm, and (d) 627 nm light sources at varying optical intensities. (e) Minimum NPC occurrence at various wavelengths. (f) Shifts in flat-band voltage (*V*_fb_) as a function of illumination intensity.

When the gate and source are shorted (*V*_G_ = 0), the Fermi levels of Si and MoS_2_ align, and the accumulated charge distributes across the entire structure, including both the bulk Si and the MoS_2_ channel. The charge distribution follows the relationship: $Q_{\mathrm{Si}}=-\frac{C_{d}}{C_{d}+C_{\mathrm{ox}}}Q_{t}$ and, $Q_{{\mathrm{MoS}}_{2}}=-\frac{C_{\mathrm{ox}}}{C_{d}+C_{\mathrm{ox}}}Q_{t}$ where *C*_d_ is the depletion capacitance, *C*_ox_ is the oxide capacitance, and *Q*_t_ represents the total accumulated charge at the Si/SiO_2_ interface.^23^

As discussed above, at low illumination intensity, the charge *Q*_t_ is negative due to electron accumulation, and the corresponding induced holes are transferred to the MoS_2_ channel, reducing the electron density and resulting in negative photocurrent. When the built-in potential is neutralized by the opposing photovoltaic effect, the hole charge reaches its maximum value. Further increasing the illumination intensity causes *Q*_t_ to become positive, signifying hole accumulation, which induces electron transfer to MoS_2_, thereby increasing electron density and leading to a gradual transition from negative to positive photocurrent.

Once the electrical connection between the gate and source electrodes is established, photogenerated carriers not trapped at the Si/SiO_2_ interface are transferred into the MoS_2_ layer, contributing to the photocurrent measured between the source and drain. This carrier injection facilitates hole transport from source to drain, resulting in negative photocurrent (NPC). An equivalent circuit diagram comparing the floating-gate and gate–source shorted configurations could be found in the Supporting Information S4. While the NPC effect has been widely reported in previous studies,^24–26^ our findings highlight the crucial role of the gate material in modulating the optoelectronic response of phototransistors.

### 2.2 Gate Contribution in MoS_2_ FET

The characteristics of negative photocurrent (NPC) are strongly influenced by the trapping mechanisms at the Si/SiO_2_ interface. Carrier trapping at this interface can be observed through the shift in the threshold voltage (*V*_th_) of the FET device, providing insight into the charge trapping dynamics. Figure 3a shows shifts in *V*_th_ of our device under various illumination intensities, with a light source of 627 nm. Upon illumination, *V*_th_ increases, suggesting electron trapping at the Si/SiO_2_ interface and leading to an increase in NPC. As illumination intensity increases, the trapping of electrons saturates and the trapping of holes dominates, thus resulting in a decrease in NPC. The point at which *V*_th_ shift = 0 is when NPC reaches its minimum, as illustrated in Figure 3b. In this figure, the photocurrent response under a 515 nm light source is shown to rapidly decrease upon illumination due to interface trapping at Si/SiO_2_, resulting in NPC. As the electron traps saturate and holes are continuously trapped, electrons are introduced into MoS_2_, leading to a reduction in NPC.

The injection of carriers from Si into MoS_2_ significantly impacts the electrical characteristics of the device. Figure 3b–d present the *I*_DS_–*V*_DS_ curves under illumination at wavelengths of 515, 590, and 627 nm, respectively, with varying optical intensities. The illumination intensity at which the minimum NPC occurs increases as the wavelength increases, as shown in Figure 3e. This trend can be attributed to the absorption characteristics of Si, as shown in Figure 2a. As the wavelength increases, the optical absorption in Si decreases, resulting in fewer photogenerated carriers. Consequently, a higher illumination intensity is required to saturate the electron traps at the Si/SiO_2_ interface. This explains why the transition to reduced NPC shifts toward higher intensities at longer wavelengths, reinforcing the role of the gate material in the observed photocurrent behavior.

The diminishing NPC effect becomes more pronounced at higher *V*_DS_, where the increased electric field further suppresses electron mobility, thereby altering the overall charge transport dynamics within the device. The variation in the photocurrent slope under different illumination intensities and *V*_DS_ conditions is directly correlated with the number of photogenerated carriers injected into the MoS_2_ layer. The slope reflects the rate at which trapped holes accumulate at the Si/SiO_2_ interface, influencing the observed photocurrent behavior. Transfer-curve data (*I*_DS_–*V*_G_) measured under a series of illumination intensities and different wavelengths are provided in the Supporting Information S5.

Figure 3b–d illustrate a clear reduction in the photocurrent slope with increasing illumination wavelength under identical *V*_DS_ and optical intensity conditions. Under the gate–source shorted condition (Figures 1c and 3b–d), the interfacial field created by trapped holes at the Si/SiO_2_ interface injects electrons into the MoS_2_ channel, increasing the channel carrier density and boosting the photocurrent by more than an order of magnitude compared with the floating-gate configuration (Figure 1d). This trend is attributed to wavelength-dependent absorption differences, further confirming that the primary source of photoinduced carriers is Si rather than MoS_2_. Notably, although MoS_2_ exhibits higher calculated absorption at 590 nm compared to 515 nm (Figure 2a), the corresponding photocurrent response remains lower at longer wavelengths. This discrepancy strongly suggests that the majority of photogenerated carriers originate from the Si gate material rather than from the MoS_2_ layer, reinforcing the role of the gate material in the observed optoelectronic response.

The photocurrent (*I*_ph_) can be expressed as the change in source–drain current (*I*_DS_) and is given by the following equation:^20^

$$I_{\mathrm{ph}}=\Delta I_{\mathrm{DS}}=-q\mu_{n}\Delta n\frac{V_{\mathrm{DS}}}{L}$$ 1

where *q* is the elementary charge and Δ*n* denotes the change in carrier concentration. The variation in carrier concentration can be related to the change in gate voltage (Δ*V*_G_) as follows:

$$\Delta n=\frac{C_{0}}{q}\Delta V_{G}$$ 2

By combining eqs 2 and 3, the equivalent change in *V*_G_ induced by illumination can be determined. The calculated Δ*V*_G_ can then be related to the shift in threshold voltage (Δ*V*_th_) by the following expression:

$$\Delta V_{G}=\Delta V_{\mathrm{th}}+\Delta V_{\mathrm{fb}}$$ 3

Where Δ*V*_fb_ represents the change in flat-band voltage. By inserting the values of Δ*V*_G_ and Δ*V*_th_, which are extracted from Figure 3a, the relationship between flat-band voltage (*V*_fb_) and illumination intensity can be established. Figure 3f illustrates this relationship, showing that *V*_fb_ increases with increasing illumination intensity, indicating the trapping nature of interface states at the Si/SiO_2_ interface.

To verify that the observed behavior is not sample-specific, an additional device with a thicker SiO_2_ gate dielectric (300 nm instead of 100 nm) was fabricated. Despite the reduced oxide capacitance, this device also exhibited clear negative photocurrent under weak illumination (see the Supporting Information, Figure S6), demonstrating that the NPC effect persists across different oxide thicknesses and remains robust against variations in gate–channel coupling.

To further clarify the role of interface traps in the observed NPC behavior, temperature-dependent *I*–*V* measurements were performed between 8 and 400 K under controlled illumination (Supporting Information, Figure S7). The magnitude of NPC decreases with decreasing temperature and becomes negligible below approximately 200 K, indicating that trap-assisted charge separation, rather than intrinsic MoS_2_ photoconduction, dominates the photoresponse. Moreover, the illumination intensity corresponding to the NPC minimum shifts toward higher values as temperature increases, consistent with thermally activated hole emission and the resulting modulation of the interfacial potential at the Si/SiO_2_ interface.

To exclude the possibility that the observed NPC originates from contact photothermoelectric/photovoltaic effects or local heating under weak illumination, spatially resolved photocurrent measurements were performed. These measurements were conducted under both gate–source shorted and floating-gate configurations (Supporting Information S8). The photocurrent obtained under the floating-gate condition remained significantly smaller than that in the shorted-gate configuration for all illumination positions, indicating that the NPC does not arise from intrinsic channel transport or contact-related thermal effects.

Trion formation is a well-established mechanism for NPC in monolayer MoS_2_ under certain excitation conditions and is typically accompanied by illumination-dependent optical signatures,^27^ most notably enhancement of the negatively charged exciton (X^–^) peak in photoluminescence (PL).^28–30^ To examine whether trions contribute to the NPC observed here, we performed PL measurements on the MoS_2_ FET under varying illumination intensity and gate-bias conditions, as provided in the Supporting Information S9. The results demonstrate that, although trions are present in monolayer MoS_2_, their population remains essentially unchanged under the ultraweak illumination and gating conditions relevant to this study, ruling out trion formation as the origin of the observed negative photocurrent.

### 2.3 Gate Contribution in Metal Film FET

To further investigate the impact of gate, the MoS_2_ layer in our device was replaced with a metal film composed of Ti (20 nm) and Au (40 nm). The detailed structure and optical microscope image can be found in the Supporting Information S10. Figure 4a presents the calculated absorbance spectra for both Si and the Au/Ti film. Unlike MoS_2_, metals exhibit strong optical absorption due to their high density of free electrons. However, photogenerated electron–hole pairs within the Au/Ti film are negligible due to the free-electron behavior of the metal. Furthermore, the high density of free electrons in the Au/Ti film results in a low electron–hole recombination efficiency, which minimizes the contribution of the photoconductive effect. This fundamental difference in electronic properties provides further insight into the role of gate material in modulating the photocurrent response of phototransistors.

**Figure 4:**
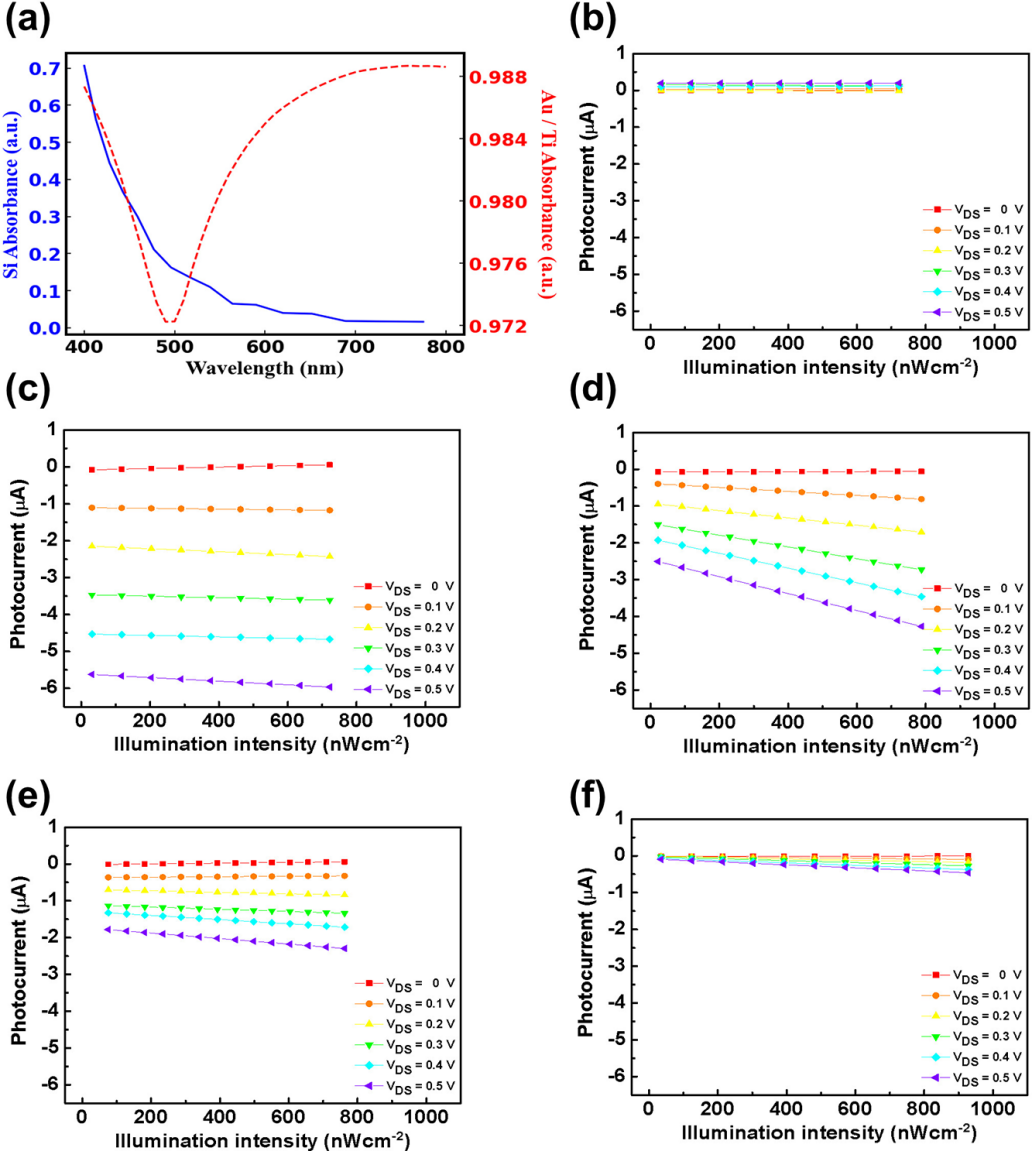
(a) Optical absorbance spectra of Si (blue curve) and Au/Ti (red curve), highlighting the differences in absorption properties. (b) Photocurrent response of the Au/Ti-based FET under illumination with a 460 nm light source at varying optical intensities, without a gate-source electrodes connection. (c–f) Photocurrent response of the Au/Ti-based FET under different *V*_DS_ values when illuminated with (c) 460 nm, (d) 515 nm, (e) 590 nm, and (f) 627 nm light sources at varying intensities, demonstrating the dependence of photocurrent generation on the Si gate electrode.

Figure 4b presents the photocurrent response of the Au/Ti/SiO_2_/Si structure under illumination with a 460 nm light source, in the absence of a gate–source electrodes connection. Similar to the results observed in Figure 1b, no significant photocurrent was detected. Figure 4c–f display the photocurrent response of the device when the gate and source electrodes are connected, under illumination with 460, 515, 590, and 627 nm light sources at varying intensities. Consistent with the results obtained in the MoS_2_ FET configuration, NPC was observed in the Au/Ti-based device. This finding further reinforces the conclusion that the primary origin of the photoinduced carriers is the Si gate electrode, rather than the sensing material itself, highlighting the critical role of the gate material in the observed photocurrent generation. Compared to MoS_2_, Au/Ti exhibits a higher optical absorption, resulting in fewer photons reaching the underlying Si layer. Consequently, the reduction in NPC is no longer observed. However, the difference in photocurrent under varying *V*_DS_ conditions becomes more pronounced. As shown in Figure 4c–f, the difference between *V*_DS_ = 0 and *V*_DS_ = 0.5 V decreases significantly with increasing wavelength. This trend can be attributed to the wavelength-dependent carrier injection from Si, where the reduced absorption at longer wavelengths leads to a lower density of photogenerated carriers in the gate material.

As calculated in Figure 4a, the optical absorbance of the Au/Ti film increases with wavelength. However, as discussed earlier, photocarrier generation within metal thin films is highly improbable due to their electronic nature. Therefore, the only viable source of photogenerated carriers is the Si gate electrode, reinforcing the conclusion that the photocurrent response originates from charge injection at the gate material, similar to the mechanism observed in the MoS_2_-based device. A comparison between calculated absorbance and measured photocurrent could be found in the Supporting Information S11.

To test the consistency of our proposed mechanism, a graphene-based FET with a structure similar to the MoS_2_ device was fabricated and tested, as presented in the Supporting Information S12. The results demonstrate that the NPC mechanism reported in this work is not specific to MoS_2_, but represents a general phenomenon applicable to thin-film-based FETs under identical gate–source shorted conditions.

## 3 Conclusions

In this study, thin-film-based FET photodetectors were investigated to elucidate the origin of photogenerated carriers. While prior research has largely attributed photocurrent generation to the PC and PV effect within the sensing layer—often neglecting the influence of the gate material—our findings reveal that the primary source of photocarriers is more closely associated with the gate material itself. The observation of NPC in our devices served as an initial indication, prompting a systematic analysis that ultimately traced the origin of the photogenerated carriers to the gate electrode. To substantiate this conclusion, optical absorption spectra of the materials used in this study were calculated and compared. In MoS_2_-based FET photodetectors, the observed variations in NPC slope and photocurrent behavior under different *V*_DS_ were consistent with the calculated absorption spectra, lending further support to our hypothesis. Moreover, measurements from metal (Au/Ti)-based FET photodetectors reinforced this conclusion, confirming that the photocurrent predominantly originates from the Si gate electrode rather than the sensing layer. These findings challenge conventional assumptions regarding photocurrent generation in thin-film FET photodetectors and underscore the critical role of the gate material, offering new insights for the design and optimization of next-generation optoelectronic devices.

## 4 Methods/Experiments

### 4.1 Sample Characteristics

The quality and thickness of the MoS_2_ layer were verified using Raman and photoluminescence (PL) spectroscopy. As shown in Figure 5a, the Raman spectrum exhibits characteristic *E*_g_^2^ and *A*_g_^1^ peaks at 385.6 cm^–1^ and 403.5 cm^–1^, respectively, with an 18 cm^–1^ separation indicative of monolayer MoS_2_.^31^ The inset Raman mapping confirms uniformity across the sample surface. Figure 5b presents the PL spectrum, acquired with a 532 nm excitation, showing a peak at 681 nm—consistent with monolayer emission and supporting the high quality of the synthesized film in agreement with established references.^32,33^

**Figure 5:**
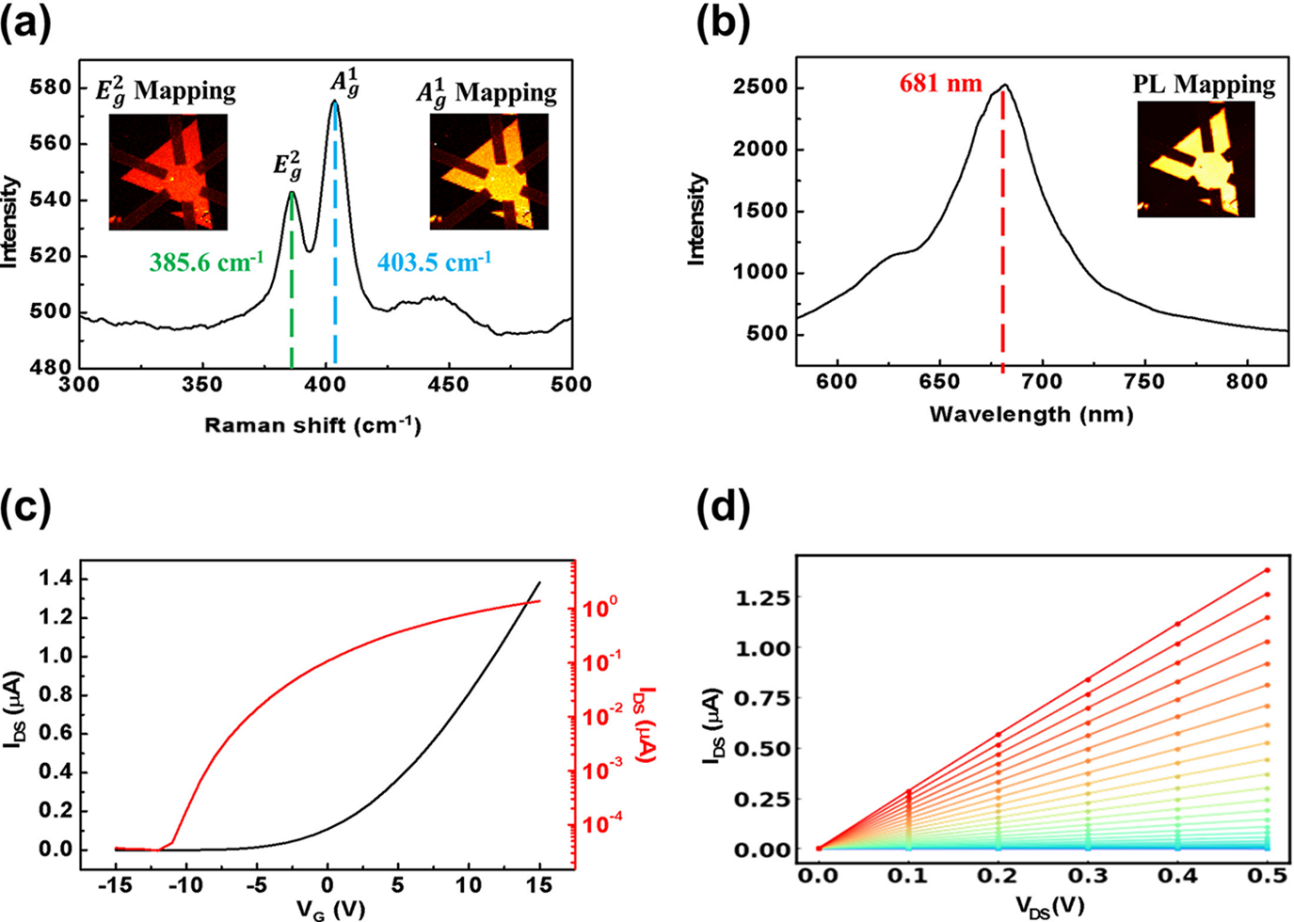
(a) Raman spectrum of the MoS_2_-based photodetector, with the inset showing Raman mapping results of the *E*_g_^2^ and *A*_g_^1^ peaks, indicating uniformity. (b) Photoluminescence (PL) spectrum of the MoS_2_-based photodetector, with the inset displaying PL mapping results, confirming homogeneity across the device. (c) *I*_DS_–*V*_G_ characteristics of the FET device at *V*_DS_ = 0.5 V under dark and vacuum conditions, shown in both linear scale (black curve) and logarithmic scale (red curve). (d) *I*_DS_–*V*_DS_ characteristics of the FET device, where the linear response suggests the formation of ohmic contacts.

To reduce interference from ambient adsorbates, all electrical measurements were performed under vacuum (see the Supporting Information S2 for setup details). Figure 5c shows the transfer curve of the device (*I*_DS_ vs *V*_G_ at *V*_DS_ = 0.5 V), demonstrating typical n-type behavior consistent with previous reports on MoS_2_ FETs.^17^ The output characteristics in Figure 5d reveal a linear *I*_DS_–*V*_DS_ relationship, indicating the formation of ohmic contacts between the metal electrodes and the MoS_2_ channel.

The carrier mobility (μ) of the fabricated device under dark and vacuum conditions was determined to be 7 cm^2^·V^–1^·s^–1^, calculated using the following expression:

$$\mu =\frac{\partial I_{\mathrm{DS}}}{\partial V_{\mathrm{GS}}}\times \frac{L}{C_{o}\cdot W\cdot V_{\mathrm{DS}}}$$ 4

where the channel length (*L*) is approximately 11 μm, and the channel width (*W*) is around 3 μm. The oxide capacitance per unit area, *C*_o_, is given by Co=εoxdox=3.36×10−8F·cm−2 where ε_ox_ represents the permittivity of SiO_2_. The extracted carrier mobility is consistent with values reported in previous studies, confirming the performance of the device.^34^

## Supplementary Material



## Notes

Detailed device fabrication procedures, experimental setup descriptions, equivalent circuit analysis of floating-gate and gate–source shorted configurations, additional transfer characteristics under varying illumination conditions, temperature-dependent photocurrent measurements, spatially resolved photocurrent mapping, graphene-based FET control experiments, photoluminescence analysis excluding trion contributions, and supplementary C–V characterization data (PDF)
