# Supplementary material for: Revealing the Active Role of the Gate Electrode in Weak-Light Detection
Source: ACS Nano. 2026 Mar 5;20(10):8266–74. doi: 10.1021/acsnano.5c13029 (PMC13001079; doi:10.1021/acsnano.5c13029)
Supplement: Supplementary file 1 [file nn5c13029_si_001.pdf]

# Supporting information

## Revealing the Active Role of the Gate Electrode in Weak-Light Detection

*Tzu-En Huang[1], Chen-Yu Wang[1], Hua-Hsing Liu[1], Bor-Wei Liang[2], Shih-Chia, Peng[3], You-Jia, Huang[3], Yann-Wen Lan[3,*], Kuan-Ming Hung[4,*], Kuang Yao Lo[1,*]*

[1]Department of Physics, National Cheng Kung University, 701 Tainan, Taiwan

[2]Institute of Electro-Optical Engineering, National Taiwan Normal University, Taipei 11677, Taiwan

[3]Department of Physics, National Taiwan Normal University, Taipei 11677, Taiwan

[4]Department of Electronic Engineering, National Kaohsiung University of Science and Technology, 807 Kaohsiung, Taiwan

## S1. Sample fabrication

Through sulfuring $MoO_3$ powders, $MoS_2$ flakes were grown by chemical vapor deposition (CVD) on C-cut $Al_2O_3$ substrates. After fabrication, $MoS_2$ sequentially went through acetone bath, isopropyl alcohol (IPA) rinse, then was submerged into N-methyl-2-pyrrolidone (NMP) for 20 minutes to remove surface residue, followed by a rinsing of IPA and dried by nitrogen gas. Poly (methyl methacrylate) (PMMA) was then spin-coated onto the sample and solidified on a heating plate. The $MoS_2$/PMMA structure was then wet transferred (ammonia solution was chosen as lifted off medium) onto a pre-fabricated substrate with array of standard pads. This substrate comprises a heavily p-doped silicon wafer, serving as the gate electrode, and a 100 nm-thick $SiO_2$ layer, functioning as the gate dielectric. After the transfer process, metal contacts (20-nm Bi/40-nm Au) were patterned using UV exposure lithography and thermal evaporation. A standard lift-off process using acetone, IPA and DI water is then carried out[1].

## S2. Experimental setup

Following the fabrication process, source, drain, gate bias were wire-bonded onto a PLCC44 platform. Subsequently, the assembled device was loaded into an airtight vacuum chamber. A source meter (Keithley 2400) was employed to measure electric properties of the phototransistor. A military connector was used to extract signals from within the vacuum chamber. A mechanical pump was applied to lower and maintain the pressure within the chamber to approximately $10^{-3}$ torr, as determined via an air pressure gauge (CVG101, InstruTech), to avoid any adsorbate interference.

## S3. Details of the smoothing procedure used in CV measurements

CV measurements were performed using an AC excitation amplitude of 5 mV, which provides a high signal-to-noise ratio while still operating within the quasi–small-signal regime for our device geometry. Although increasing the AC amplitude can further suppress electronic noise, amplitudes that are too large are known to introduce nonlinear averaging effects that broaden the CV response and shift the apparent threshold voltage[2]. Therefore, 5 mV was chosen as an optimal compromise that minimizes noise while avoiding measurement-induced distortion. The raw CV curves (Figure S3a) exhibit illumination-dependent features that remain stable and repeatable across measurements. Savitzky–Golay filter (2nd-order polynomial, 11-point window) was applied to the raw data as shown in Figure 3e. The same fine structure remains after smoothing, indicating that the observed features are intrinsic and not artifacts of measurement noise.

To further confirm this phenomenon, we performed additional CV measurements on a separate device under multiple probe frequencies (1, 2, 4, 8, and 10 kHz), again using an AC amplitude of 5 mV. Figure S3b shows that all frequencies exhibit the same evolution and the same characteristic NPC-related maximum CV shift

near ~63 nWcm$^{-2}$. The frequency-independent persistence of these features demonstrates that they originate from interface-trap dispersion at the $Si/SiO_2$ depletion region, not from random noise.

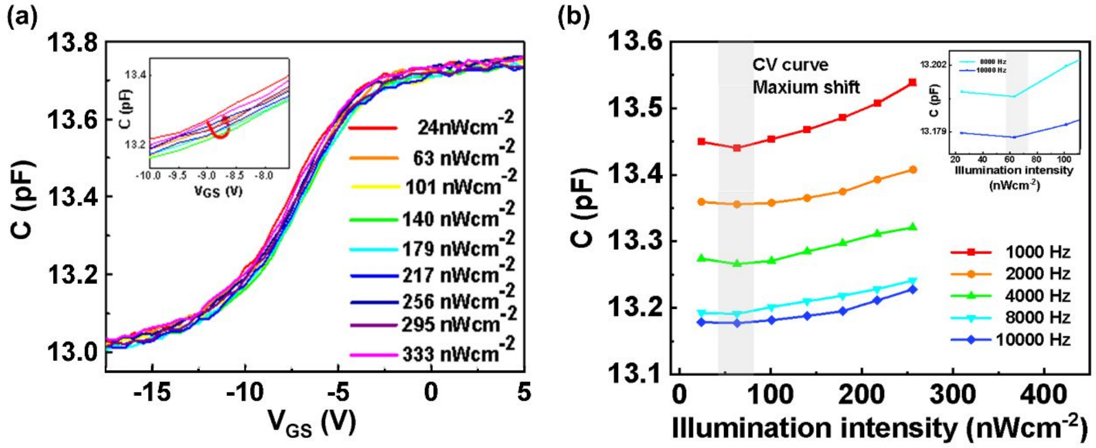


Figure S3. (a) Raw CV curves under different illumination intensities (590 nm). (b) Illumination-dependent CV shift measured at multiple probe frequencies (1–10 kHz), all showing the same NPC-related maximum near ~63 nWcm$^{-2}$.

## S4. Equivalent circuit diagram of floating-gate and gate–source shorted configurations

To clarify how the gate–source short modifies the electrostatic boundary conditions compared to the floating-gate configuration, the equivalent circuits illustrating the paths for photocarrier injection across the oxide are shown in Figure S4. In both cases, the $MoS_2$ channel is represented by a channel resistance. The oxide capacitance ($C_{OX}$) connects the channel to the Si substrate, which includes a depletion capacitance ($C_d$) and serves as the primary region for photocarrier generation.

(a) Gate–source shorted configuration ($V_G$ = 0 V):

The gate node is tied to the source, fixing the potential of the Si gate relative to the $MoS_2$ channel. Under illumination, photogenerated carriers appear across the $C_{OX}$–Cd network, and this electrical connection enables charge injection from the substrate into the $MoS_2$ layer. The resulting capacitive coupling and trap-assisted field modulation explain the observed negative-photocurrent (NPC) behavior and its dependence on illumination intensity.

(b) Floating-gate configuration:

The gate electrode is electrically isolated, and no electrical conduction path exists between the substrate and the $MoS_2$ channel. Photogenerated carriers in Si cannot drive charge transfer through $C_{OX}$, only displacement currents occur. Consequently, the $MoS_2$ channel responds primarily to its intrinsic photoconductivity, and the gate-induced photoresponse is suppressed.

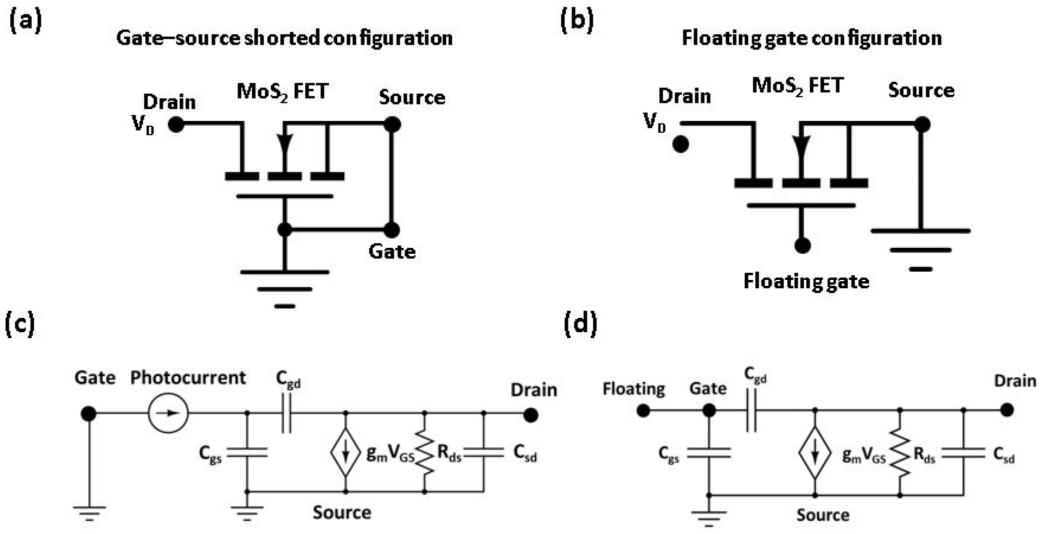


Figure S4. (a, b) Device configurations for the $MoS_2$ phototransistor under (a) gate–source shorted and (b) floating-gate conditions. (c, d) Corresponding small-signal equivalent circuit models.

## S5. Transfer-curves ($I_{DS}$–$V_G$) under a various illumination intensities and different wavelengths

Figure S5 presents the transfer characteristics ($I_{DS}$–$V_G$) of the $MoS_2$ phototransistor measured under a series of illumination intensities and different wavelengths. The curves exhibit a clear evolution in both threshold voltage ($V_{th}$) and photocurrent magnitude as the light power increases.

At low illumination levels, $V_{th}$ shifts toward more positive values as electron trapping at the Si/$SiO_2$ interface dominates. With increasing illumination intensity, hole trapping becomes significant, leading to a reversal of the interfacial potential and a shift of $V_{th}$ back toward negative values.

The photocurrent magnitude follows a similar trend—initially increasing in the negative direction as trap-assisted charge separation strengthens, then decreasing as the interfacial potential reverses. This systematic and reversible behavior confirms that the photoresponse is governed by trap-assisted charge transfer processes at the Si/$SiO_2$ interface, rather than intrinsic $MoS_2$ channel effects.

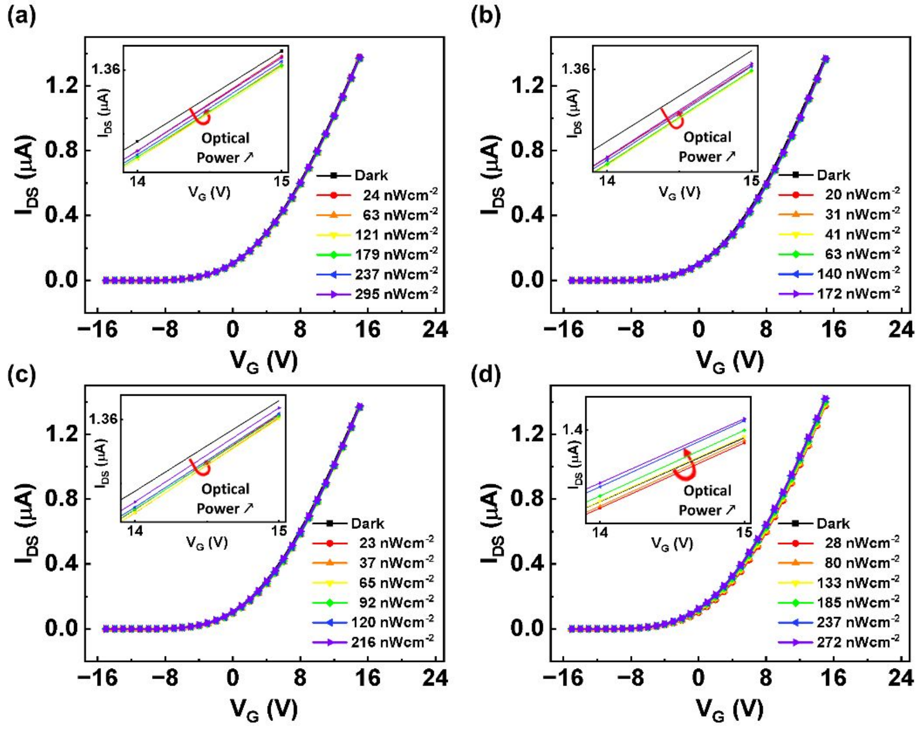


Figure S5. Transfer characteristics ($I_{DS}$–$V_G$) of the $MoS_2$ phototransistor under different illumination intensities for wavelengths of (a) 627 nm, (b) 590 nm, (c) 515 nm, and (d) 460 nm. The insets highlight the characteristic first decrease and subsequent recovery of photocurrent with increasing optical power.

## S6. NPC repeatability across different oxide thickness

To examine whether the negative photocurrent (NPC) behavior is sample-specific, we measured a device with the same geometry as the main-text sample but with a 300 nm $SiO_2$ gate dielectric (versus 100 nm in the primary device). As shown in Figure S6, the current–illumination characteristics (with parameters $V_{DS}$ = 0.5 V and $V_{GS}$ = 0 V) exhibit the same weak-illumination NPC behavior observed in the main device. Although the thicker oxide reduces $C_{OX}$ and therefore weakens gate–channel coupling, the presence of clear NPC confirms that the effect is not sample-specific and is governed primarily by interface-trap dynamics. This demonstrates that NPC remains robust across oxide stacks with different thicknesses.

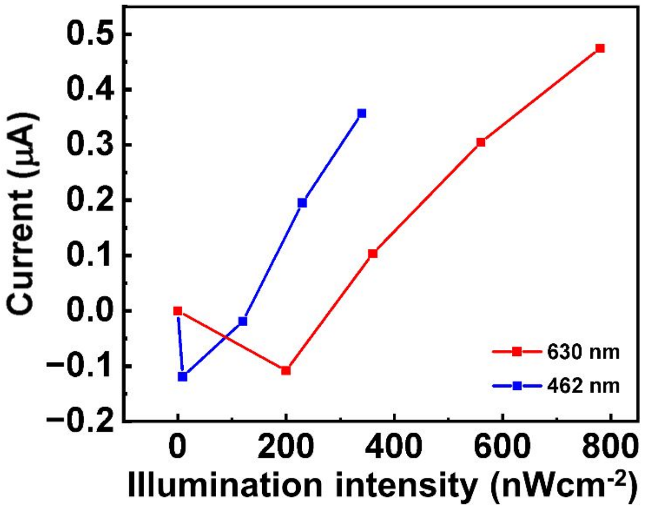


Figure S6. Current–illumination intensity relationship for the device with a 300 nm $SiO_2$ gate dielectric. Negative photocurrent (NPC) emerges under weak illumination.

## S7. Temperature-dependent I–V measurements

To clarify the role of interface traps in the observed negative-photocurrent (NPC) behavior, temperature-dependent I–V measurements were performed between 8 K and 400 K under controlled illumination (Figure S7). The magnitude of NPC decreases as temperature decreases and becomes negligible below approximately 200 K, indicating that trap-assisted charge separation—rather than intrinsic $MoS_2$ photoconduction—dominates the photoresponse. At low temperature, hole emission from $Si/SiO_2$ interface traps is strongly suppressed, weakening the built-in field and reducing electron injection into $MoS_2$[3, 4]. As temperature increases, the illumination intensity corresponding to the NPC minimum shifts toward higher values, because enhanced thermal energy accelerates hole emission and decreases steady-state trap occupancy. Consequently, stronger illumination is required to repopulate traps and re-establish the interfacial field that counteracts the photovoltaic potential, shifting the NPC minimum to higher intensity.

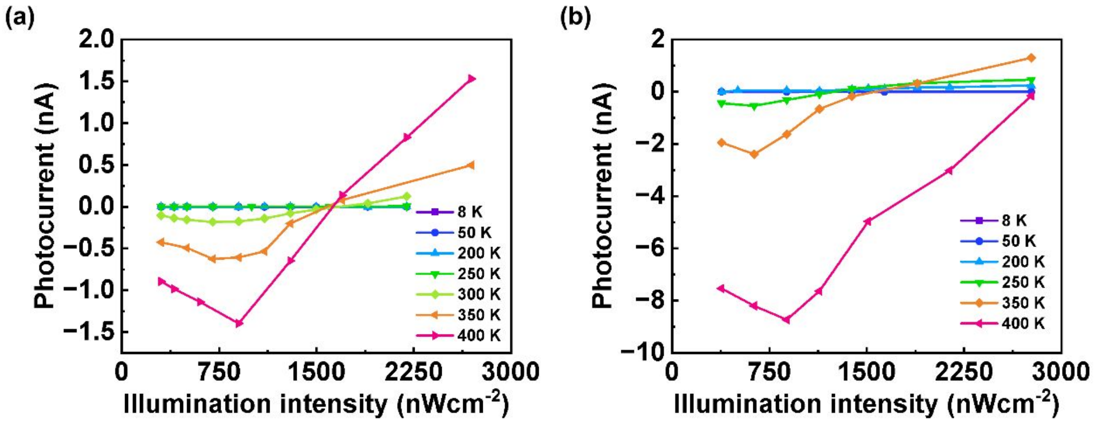


Figure S7. Temperature-dependent photocurrent under (a) 515 nm and (b) 460 nm illumination. NPC weakens at low temperature and vanishes below ~200 K, while the NPC minimum shifts to higher illumination intensity with increasing temperature, reflecting thermally activated trap dynamics.

## S8. Spatially Resolved Photocurrent Measurements

To distinguish substrate-induced photocurrent from contact photothermoelectric or photovoltaic contributions, spatially resolved photocurrent measurements were performed using a focused 532 nm laser. The laser spot (~1.5 μm) was positioned at three locations on the $MoS_2$ phototransistor: (i) entirely on the channel, (ii) half on the channel and half on the electrode, and (iii) entirely on the electrode, as shown in Figure S8.

Measurements were conducted under both (i) gate–source shorted and (ii) floating-gate configurations. The resulting photocurrent–illumination characteristics (Figure S8a) show that under the gate–source shorted condition, the device exhibits a clear negative photocurrent (NPC) and NPC reversal behavior. In contrast, under the floating-gate condition (Figure S8b), the photocurrent remains extremely small and positive for all illumination positions.

These results demonstrate that contact photothermoelectric or photovoltaic effects do not contribute to the NPC. Although small contact-related signals exist, they are more than three orders of magnitude weaker than the NPC arising from substrate-mediated charge transfer through the $Si/SiO_2$ interface.

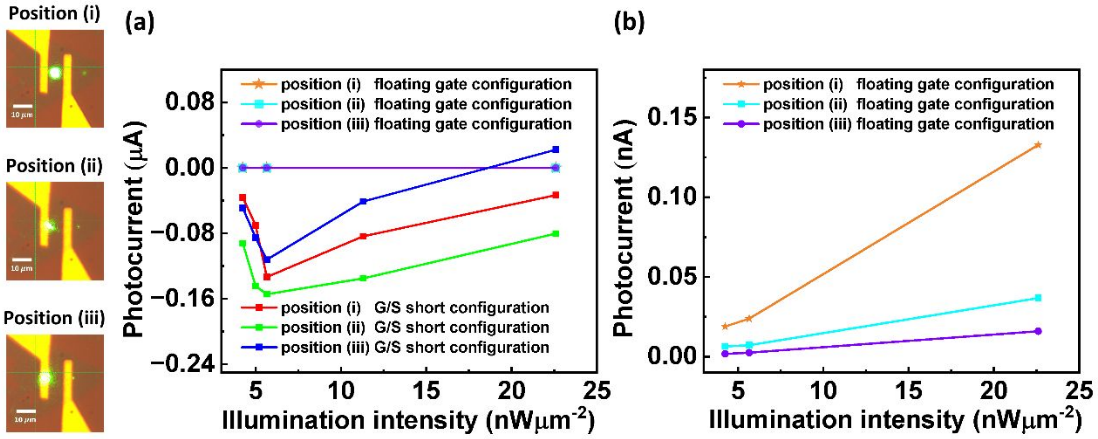


Figure S8. Spatially resolved photocurrent measurements at (i) channel, (ii) channel/electrode boundary, and (iii) electrode. (a) Both gate–source shorted configuration and floating-gate configuration. (b) Rescaled floating-gate configuration.

## S9. Photoluminescence Analysis and Trion Exclusion

Trion formation is a well-established mechanism for negative photocurrent (NPC) in monolayer $MoS_2$ under certain excitation conditions[5] and is typically accompanied by illumination-dependent optical signatures, most notably enhancement of the negatively charged exciton ($X^-$) peak in photoluminescence (PL)[6-8]. To evaluate whether trion formation contributes to the NPC observed in this study, PL measurements were performed on the $MoS_2$ FET under varying illumination intensity and gate-bias conditions using a 532 nm excitation source at room temperature (Figure S9a).

The PL spectra were fitted with three Lorentzian components corresponding to the A exciton (~676 nm), B exciton (~621 nm), and $X^-$ (~689 nm), as shown in Figure S9b. The extracted peak intensities (Figure S9c) reveal that while the A exciton intensity increases strongly with illumination, the B exciton and $X^-$ components remain weak and show negligible growth even at illumination intensities up to 3.7 mW. This indicates that the trion population is not significantly enhanced under the experimental conditions relevant to the NPC regime, which corresponds to ultra-weak illumination levels far below those typically required for trion-dominated PL responses[9-10].

PL spectra were also acquired under different gate-voltage ($V_{GS}$) conditions,

including floating-gate operation. As shown in Figure S9d, the fitted intensities of the A, B, and X⁻ peaks remain essentially unchanged with gate bias or gate connectivity, demonstrating that trion population is insensitive to electrostatic gating in this device. Together, these results provide direct optical evidence that trion formation does not contribute significantly to the negative photocurrent observed in this study.

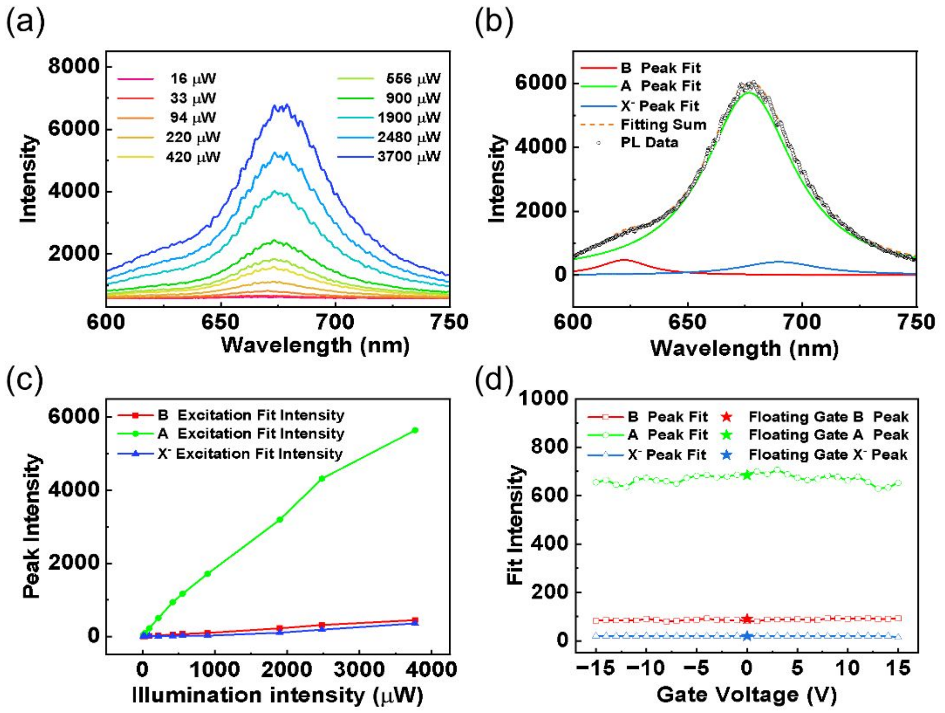


Figure S9. (a) PL spectra of the $MoS_2$ FET under increasing illumination intensity. (b) PL spectrum fitted using three Lorentzian components corresponding to A exciton, B exciton, and negatively charged exciton $X^-$, the dash line represents the total fitting curve. (c) Peak intensities of A, B, and $X^-$ excitations as a function of illumination intensity. (d) Peak intensities of A, B, and $X^-$ excitations as a function of gate voltage under both gate-biased and floating-gate conditions.

## S10. Au/Ti sample fabrication

A 20 nm thick layer of titanium (Ti) and a 40 nm thick layer of gold (Au) were deposited on 100 nm of silicon dioxide ($SiO_2$), with aluminum wires wire-bonded on top of the Au layer to serve as the source and drain electrodes. Figure S10 shows the schematic structure of the device, along with an optical microscope image for visualization.

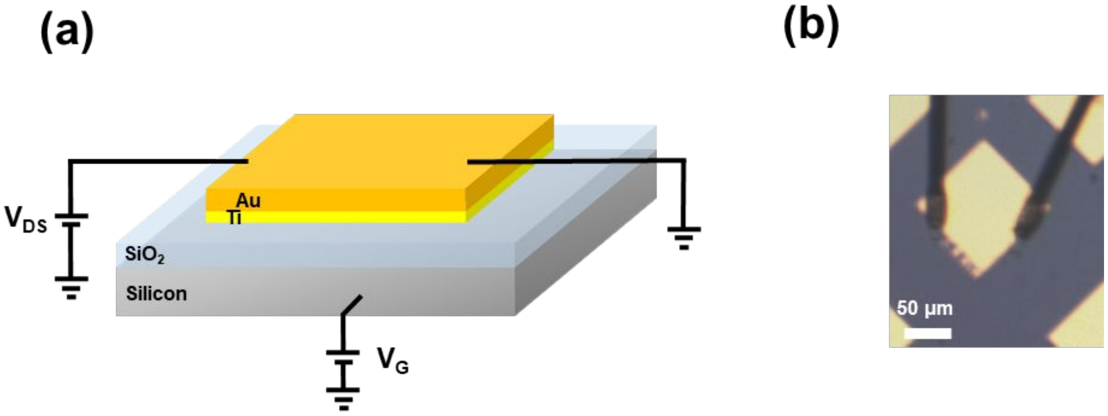


Figure S10. (a) Schematic structure of the Au/Ti/$SiO_2$/Si sample. (b) Optical microscope image of the Au/Ti/$SiO_2$/Si sample.

## S11. Comparison between calculated absorbance and measured photocurrent

The calculated absorbance spectra of Si (blue curve) and Au/Ti (red curve), is shown in Figure S11a. Figure S11b presents the absolute photocurrent extracted from Figures 5c-5d at $V_{DS} = 0.5$ V under constant illumination intensity (200 $nWcm^{-2}$). Since the photocurrent originates primarily from the gate material, its wavelength-dependent trend follows that of the Si absorbance—decreasing with increasing wavelength—further confirming the gate's dominant contribution to the photoresponse.

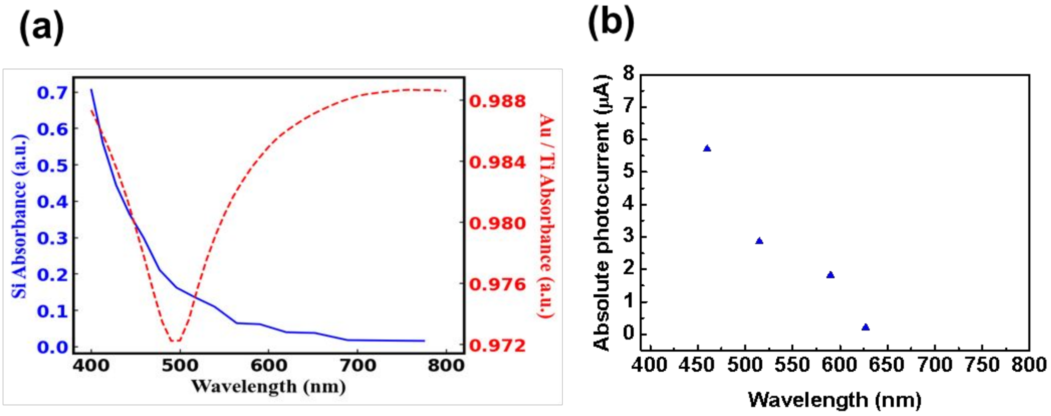


Figure S11. (a) Calculated absorbance spectra of Si (blue) and Au/Ti (red). (b) Absolute photocurrent (extracted from Figures 5c-5d) at $V_{DS} = 0.5$ V and fixed illumination intensity of 200 $nWcm^{-2}$.

## S12. Graphene-Based FET Experiment

To test the consistency of our proposed mechanism, a graphene-based FET with a structure similar to the $MoS_2$ device was fabricated, the schematic illustration of the graphene FET and an optical microscope image of the device are shown in Figures S12a and S12b, respectively.

When the gate is electrically connected to the source, optical illumination primarily generates electron–hole pairs in the Si substrate rather than in the graphene channel, owing to the minimal optical absorption of graphene. As in the $MoS_2$ and Au/Ti devices, photogenerated holes are trapped at the Si/$SiO_2$ interface, producing a light-induced interfacial electric field. This field modulates the carrier density in the graphene channel through the oxide capacitance, resulting in a change in channel conductance. Unlike monolayer $MoS_2$, which has a direct bandgap of ~1.8 eV, graphene is a gapless, high-mobility conductor with a high intrinsic carrier concentration (Figure S12c), similar to the Au/Ti device, the high density of free electrons results in a low electron–hole recombination efficiency, which minimizes the contribution of the photoconductive effect.

Figure S12d shows the photocurrent as a function of illumination intensity under a 532 nm light source. A clear NPC is observed when the gate and source are shorted,

whereas the NPC vanishes when the gate–source connection is removed. These results demonstrate that the NPC mechanism reported in this work is not specific to $MoS_2$, but is a general phenomenon applicable to film-based FETs under identical gate–source shorted conditions.

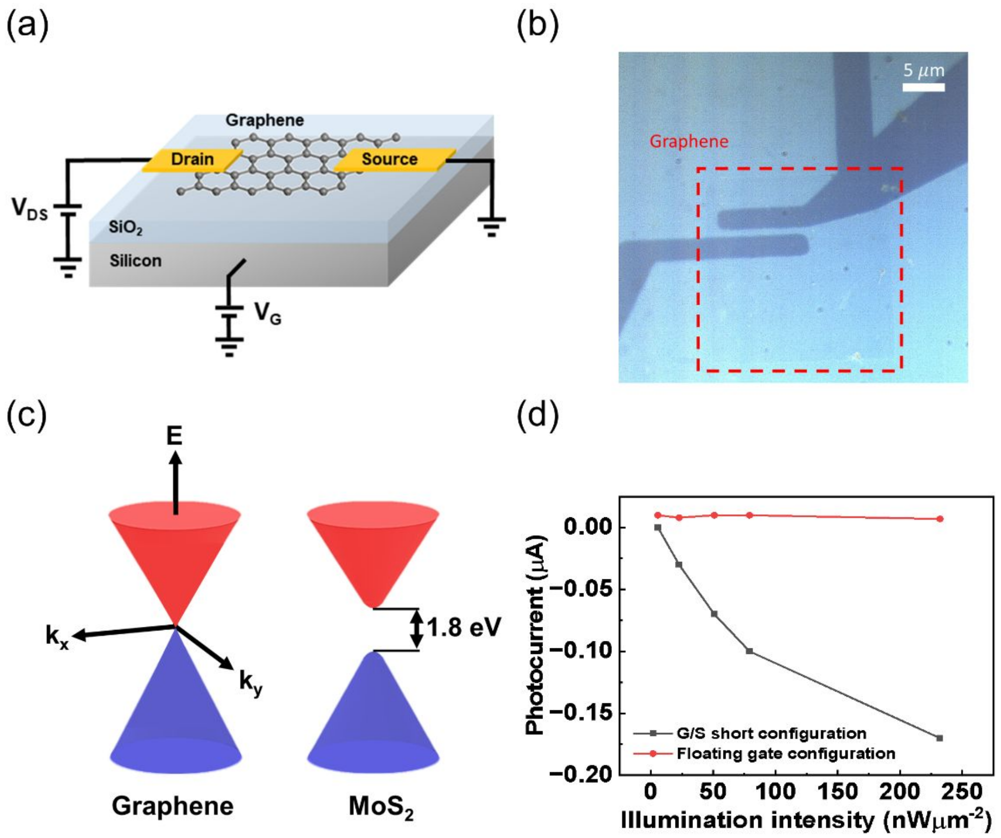


Figure S12. (a) Schematic illustration of the graphene FET. (b) Optical microscope image of the graphene device. (c) Schematic comparison of the electronic band structures of graphene (gapless Dirac cones) and monolayer $MoS_2$ (direct bandgap ~1.8 eV). (d) Photocurrent as a function of illumination intensity (532 nm) for the graphene FET under gate–source shorted and floating-gate configurations.

## Reference

[1] P.-C. Chen, C.-P. Lin, C.-J. Hong, C.-H. Yang, Y.-Y. Lin, M.-Y. Li, L.-J. Li, T.-Y. Yu, C.-J. Su, K.-S. Li, Y.-L. Zhong, T.-H. Hou, and Y.-W. Lan, “Effective N-methyl-2-pyrrolidone wet cleaning for fabricating high-performance monolayer MoS2 transistors,” Nano Res. **12**(2), 303–308 (2019).

[2] R. L. Bunch and S. Raman, "Large-signal analysis of MOS varactors in CMOS -G/sub m/ LC VCOs," IEEE Journal of Solid-State Circuits, 38, 8, 1325-1332, (2003)

[3] V.S Lysenko, I.P Tyagulski, Y.V Gomeniuk, I.N Osiyuk, "Effect of oxide–semiconductor interface traps on low-temperature operation of MOSFETs," Microelectronics Reliability, 40 (4–5), 735-738 (2000).

[4] F Rahmoune, D Bauza, “Si–SiO2 interface trap capture properties ,” Microelectronic Engineering, 59, (1–4), 115-118, (2001)

[5] Mak, K.; He, K.; Lee, C.; Lee, H.; Hone, J.; Heinz, T.; Shan and J.; Tightly bound trions in monolayer MoS2. Nature Mater 2013, 12, 207–211.

[6] Irfan, I.; Golovynskyi, S.; Bosi, m.; Seravalli, L.; Yeshchenko, O.; Xue, B.; Dong, D.; Lin, Y.; Qiu, R.; Li, B.; and Qu, J; Enhancement of Raman Scattering and Exciton/Trion Photoluminescence of Monolayer and Few-Layer MoS2 by Ag Nanoprisms and Nanoparticles: Shape and Size Effects. The Journal of Physical Chemistry C 2021,7, 125, 4119-4132.

[7] Lin, Y.; Ling, X.; Yu, L.; Huang, S.; Hsu,A.; Lee, Y.; Kong, J.; Dresselhaus, M and Palacios, T.; Dielectric Screening of Excitons and Trions in Single-Layer MoS2. Nano Letters 2014 14 (10), 5569-5576.

[8] Golovynskyi, S.; Datsenko, O.; Dong, D.; Lin, Y.; Irfan, I.; Li, B.; Lin, D and Qu, J.; Trion Binding Energy Variation on Photoluminescence Excitation Energy and Power during Direct to Indirect Bandgap Crossover in Monolayer and Few-Layer MoS2. The Journal of Physical Chemistry C 2021 125 (32), 17806-17819

[9] Golovynskyi, S.; Irfan, I.; Bosi, M.; Seravalli, L.; Datsenko, O.; Golovynska, L.; Li, B.; Lin, D.; Qu, J.; Exciton and trion in few-layer MoS2: Thickness- and temperature-dependent photoluminescence.Applied Surface Science, Volume 515, 2020, 146033, ISSN 0169-4332,

[10] Goswami, T.; Rani, R.; Hazra, K.; and Ghosh, H.; Ultrafast Carrier Dynamics of the Exciton and Trion in MoS2 Monolayers Followed by Dissociation Dynamics in Au@MoS2 2D Heterointerfaces. The Journal of Physical Chemistry Letters 2019 10 (11), 3057-3063
